# Profiles of registrant dentists and policy directions from 2000 to 2020

**DOI:** 10.1038/s41405-020-00054-1

**Published:** 2020-11-28

**Authors:** Latha S. Davda, David R. Radford, Sasha Scambler, Jennifer E. Gallagher

**Affiliations:** 1 University of Portsmouth Dental Academy, Portsmouth, PO1 2QG UK; 2 Faculty of Dentistry, Oral & Craniofacial Sciences, Centre for Host Microbiome Interactions, King’s College London,, Denmark Hill Campus, London, SE5 9RS UK

**Keywords:** Health care, Dental public health

## Abstract

**Introduction:**

The National Health Service’s reliance on overseas doctors and nurses, unlike dentists, has been widely reported. As the United Kingdom (UK) leaves the European Union, an understanding of the migration trends and possible influences are important to inform future planning.

**Aim:**

To examine trends in the profile of UK registered dentists in the context of key events and policy changes from 2000 to 2020.

**Method:**

Data were obtained from the General Dental Council via annual reports, and under ‘freedom of information’ communications; details of policy initiatives were obtained from the government and professional websites.

**Results:**

Over a 20-year period (2000–2019), the number of registered dentists increased from 31,325 to 42,469, a net increase of 36% (11,144 dentists), the majority of whom were international graduates (58%; *n* = 6,416) such that by December 2019, 28% of all registered dentists had qualified outside of the UK. Similarly, regarding new registrants, there were increases of graduates from UK (18%), EEA countries (214%) and, via the Overseas Registration Examination route (621%); and a decrease from countries with bilateral agreements for recognition (43%), in line with changes in health and immigration policies.

**Conclusions:**

International dental graduates increasingly contribute to the UK dental workforce and there is an urgent need for research into dentist migration and retention in the UK in support of patient access to dental care.

**Impact:**

- The United Kingdom (UK) dental workforce is increasingly reliant on international dental graduates representing 28% of current registrants compared with 18% in 2000.
- Health policies and immigration policies were the main drivers that influenced dental workforce migration to the UK along with wider events, such as EU expansions, global recession and Brexit.
- Pre-existing lack of research into dental workforce could add to the uncertainties of post COVID-19 oral health care access and delivery.

## Introduction

International dental graduates (IDGs) are the dentists who have qualified outside the United Kingdom (UK) and migrated to the UK with the intention to work in the profession. It is well recognised that the National Health Service (NHS) is reliant on overseas health professionals and the UK is an attractive destination country for overseas qualified doctors and nurses.^1,2^ However, little is known about the extent and the type of migration of the IDGs over the past two decades and the UK’s reliance on IDGs.

The increase in migration of dentists from Europe to the UK,^3–5^ factors that may affect their performance^6^ and the impact of Brexit on the migration of European Economic Area (EEA) dentists have been reported.^7^ However, the trends of the profiles of all the registrants over the last two decades and the policy directions that may have influenced these changes have not been correlated or reported.

Migration of IDGs to the UK is not a new phenomenon. As early as 1878, dentists who qualified in the USA, registered with the General Council of Medical Education and Registration to work in the UK much before the NHS was established.^8,9^ The establishment of NHS in 1948 meant that the responsibility to provide the basic “right to health” to its citizens rested with the state.^10^ The change in system resulted in an exodus of UK trained health professionals, reluctant to work in NHS, which led to active recruitment of the doctors trained in India and nurses trained in the Caribbean in the 1950s and 1960s;^11^ setting a precedence for active recruitment of an international health workforce.

Internationally, migration of IDGs across Europe and to Australia has caused concerns about increasing oral health inequalities in source countries, the loss of skilled dental workforce due to the inability of IDGs to integrate into the profession in destination countries, and the rights of the migrant dentists.^12,13^ Migration of dentists from low and middle income countries could deplete the oral health workforce from these countries contradicting the World Health Organisation’s Code of Conduct on International Recruitment of Health Personnel (WHO Code).^14,15^ Migrant health professionals have to overcome barriers of immigration, professional registration examinations, English language tests, financial and social difficulties in order to start practising in the destination country.^16,17^ At the same time, professional bodies responsible for patient safety must ensure that the registrants are fit for purpose.^18^

Oral health care services are only as effective as the workforce that delivers the care and they should be competent and in sufficient number to meet the needs of the population.^19^ The reliance of the NHS on overseas doctors and nurses is well documented unlike the reliance on dentists. Despite the overall increase in registered dentists, there are reports of NHS dental practices closing down due to difficulty recruiting dentists in some areas of the country.^20^ The reasons for workforce deficiency or its maldistribution are complex and may be more recently linked to Brexit; however, increasing the number of IDGs could be one of the many solutions.^21^ Therefore the aim of this study was to examine trends in the profile of UK registered dentists in the context of key events and analyse the policy changes from 2000 to 2019/2020.

## Methods

Dentist registrant data were obtained from the General Dental Council (GDC) Annual reports (2000–2017) and through ‘Freedom of Information' (FOI) requests to gain missing data and the latest figures (2000–2019). The beginning of the millennium and the first ‘Primary dental workforce review’,^22^ was selected as the starting point for data collection ending with the UK formally leaving the EU on 31st January 2020 (2000–2019/20).

The timelines of events and policy changes in health care, dentistry and immigration were obtained from websites and archives of the UK Department of Health, the Home Office, the British Dental Association, the European Union and the Organisation for Economic and Commercial Development. In the light of the past policy directions, a descriptive, ecological analysis was undertaken for the past two decades as to trends in total registrants, new registrants, net loss or gain of registrants and gender variation based on their route to registration.

## Results

### Trends in total registrants

**Fig. 1:**
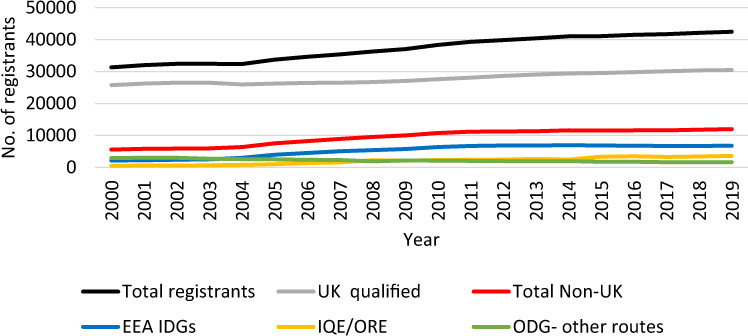
Total GDC registrants (dentists) by the route to registration. The number of GDC registered dentists increased from 31,325 to 42,469, over the 20-year period examined (2000–2019) which represents a net increase of 36% (11,144 dentists); over half of whom were IDGs (58%; *n* = 6,416). Among non-UK qualified registrants, EEA dentists were the largest contributors at 6,761 (56%) followed by IQE/ORE route 3,591 (30%) and dentists registering through other routes 1,633 (14%). The red line is the total of blue, yellow and green lines. Source: GDC annual reports and FOI requests by LD.

The GDC maintains a register of dentists based on their route to registration as UK qualified dentists, EEA qualified dentists, non-EEA qualified dentists (IQE/ORE route) and as overseas dental graduates mainly consisting of those qualifying from countries with whom UK has bilateral agreements. The number of GDC registered dentists increased from 31,325 to 42,469, over the 20-year period examined (2000–2019) which represents a net increase of 36% (11,144 dentists); over half of whom were IDGs (58%; *n* = 6,416). By December 2019, IDGs constituted 28% (*n* = 11,985) of all registered dentists (Fig. 1). Among non-UK qualified registrants, EEA dentists were the largest contributors at 6,761 (56%) followed by IQE/ORE route 3,591 (30%) and dentists registering through other routes 1,633 (14%).

This change over the two decades represented an 18% increase in UK graduates (*n* = 4,728) on the register and a 214% increase of EEA IDGs (*n* = 4606). Entrants via the overseas registration examination (IQE/ORE) route made a major contribution increasing by 621% (*n* = 3,093) and contrastingly there was a decrease of 43% (*n* = 1,633) of dentists qualifying from countries with whom the UK had bilateral agreements. 

**Fig. 2:**
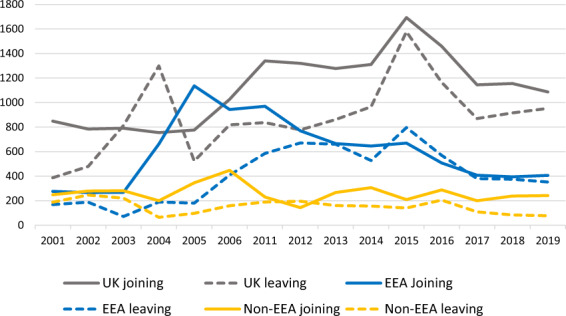
UK, EEA and non-EEA qualified dentists joining and leaving the GDC register by route to registration, 2001–2019. The total number of dentists joining the register has always been more than those leaving except in 2004, when a large number of UK qualified dentists (*n* = 1,299) left the register, the shortfall compensated mostly by EEA qualified dentists. EEA dentists leaving the register has been on the rise since 2005 and exceeded those joining in 2015 and 2016. The number of non-EEA qualified dentists leaving the register is low except in 2012. Between 2007 and 2010 data provided showed inconsistencies due to the changes in the GDC data management systems and were therefore omitted from the above graphs. Source: GDC annual reports and FOI requests by LD.

During a 7-year period (2004–2010), the percentage of newly qualified UK dentists joining the register was notably lower (34–48%) than those who qualified from outside the UK (52–66%), mainly from the EEA countries (Fig. 2); thereby resulting in a very small increase to no change to the total UK qualified registrants compared to the non-UK qualified registrants (Fig. 1). The proportion of EEA graduates joining the register fluctuated with the highest number 50% (*n* = 1,136) registered in 2005. This fell to 23% (*n* = 409) of new entrants in 2019. Entrants via the IQE/ORE route increased from 4% to 11% of registrants during the same time, whilst those from bilateral agreements/other routes have decreased from 14% to 3%. The number of entrants from IQE/ORE routes increased from 2003 onwards, with peaks in 2006, 2014, 2016 and 2019 (Fig. 2).

### Net loss or gain of dentists

The total number of dentists joining the register has always been more than those leaving, with the exception of 2004, when a large number of UK qualified dentists (*n* = 1,299) left the register; this shortfall was mostly compensated for by EEA dentists (Fig. 2). The net stock of UK qualified dentists was negative in 2005 and shows two distinct peaks of high numbers leaving in 2004 (*n* = 1,299) and 2015 (*n* = 1,578).

The EEA qualified dentists joining peaked in 2007, however, the numbers leaving the register has risen since 2005 and exceeded those joining in 2015 and 2016. The overall numbers through this route is decreasing. Analysis by country shows that the IDG numbers from some EEA countries, such as Romania, Spain, Portugal, Hungary, Bulgaria, Czech Republic and Lithuania have increased since 2015 (Table 1). The number of non-EEA dentists leaving the register was low except in 2012 thereby gradually increasing their net stock on the register.

**Table 1:** Total number of dentists who qualified from EEA countries registering in the UK from 2015 to 2019.

Year	2015	2016	2017	2018	2019
Romania	625	666	658	693	732
Spain	683	674	692	685	732
Portugal	507	496	500	494	504
Hungary	341	376	388	408	415
Bulgaria	320	308	316	323	342
Czech Republic	164	176	199	217	231
Lithuania	191	190	190	199	205
Poland	803	792	776	759	743
Greece	671	679	680	688	682
Sweden	770	738	713	694	672
Ireland	661	625	582	568	555
Germany	317	305	286	266	257
Italy	275	266	234	222	221
Denmark	115	111	108	105	100
Slovakia	46	45	52	64	68
Latvia	63	60	58	63	65
France	92	82	80	77	65
Netherlands	37	37	39	38	38
Belgium	33	31	33	33	30
Norway	27	29	30	30	29
Malta	28	27	29	29	27
Finland	22	20	20	17	17
Estonia	15	15	16	15	14
Switzerland	6	5	6	4	5

There is an overall decrease in the total registrants from the EEA countries from 2015, although flows from some countries have remained high.

Source: GDC FOI request by LD.

### Trends in gender of registered dentists, 2000–2019

**Fig. 3:**
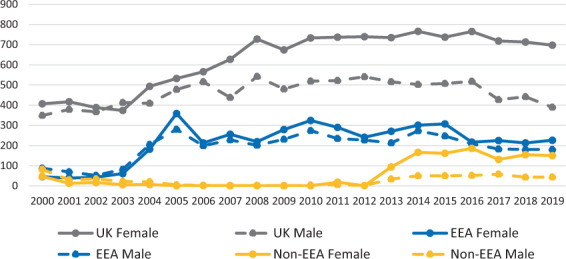
Gender analysis of the new registrants who are UK qualified, EEA qualified and non-EEA qualified, 2000–2019. The number of UK qualified female dentists has always been higher than male except in the year 2003 and the difference has increased. During 2000–2004, there were more male EEA and non-EEA qualified dentists, a trend which has subsequently reversed, with marked increase in females registering in the non-EEA group. Source: GDC annual reports and FOI requests by LD.

The proportion of female registrants has increased in all groups during the past two decades (Fig. 3). The gender gap has increased among the UK graduates after 2004. Prior to 2004, there were more male than female IDGs from EEA and non-EEA countries joining the register. This trend reversed after 2005, more notably among non-EEA dentists since 2012. By December 2019, 77% of non-EEA IDGs, 64% of UK graduates, and 56% of EEA IDGs joining the register were female.

### Trends in health policies

In dentistry, ‘Modernising NHS dentistry’^23^ led to the first review of the workforce in Primary Dental Care,^22^ highlighting the need for more dentists in England (Table 2). Historical bilateral agreements with Australia, Hong Kong, Malaysia, New Zealand, Singapore and South Africa were drawing to an end at the same time as the GDC stopped recognising degrees from these countries (2001–2005).^4^ In addition, devolution 2001, resulted in health care planning and delivery being devolved to the four nations along with the workforce planning.^24^ ‘Reform with a bite’ in England, ‘Route to reform: a strategy for primary dental care in Wales’, ‘Action plan for improving oral health and modernising NHS dental services in Scotland’ (2005) set out the means by how the dental workforce could be reformed and expanded.^25–27^ Northern Ireland’s devolved government set up Department of Health, Social Services and Public Safety (2009) to invest in dental services, which prompted workforce review.^28^ One of the strategies was to recruit more IDGs from the EEA and facilitate the entry of non-EEA dentists through an International Qualifying Examination (IQE—2001)^29^ and its replacement Overseas Registration Examination (ORE—2007).^30^ Entry for overseas dentists was further enhanced with the recognition of the License in Dental Surgery (LDS) of the Royal College of Surgeons (RCS) of England, as equivalent to the ORE in 2010, facilitating registration of additional non-EEA qualified dentists, who are recorded as coming through the ORE route by the GDC.^31^

**Table 2:** Timeline of key events in dentistry and health care in the UK affecting dental profession.

Year	Event reference	Impact on the dental profession
1948	National Health Service introduced. National Health Service Act 1946 (9 and 10 Geo. c. 81)^10^	Provision of free dental health care for all in the UK.
1950–1960	Recruitment of overseas qualified doctors (India), nurses (Caribbean (Simpson et al.))^11^	Set the precedence for international health professionals’ recruitment in UK.
1956	General Dental Council established (General Dental Council)^51^	Regulating body for the dental profession which was required to maintain a register of dental professionals permitted to work in the country.
2000	Modernising NHS Dentistry- Implementing the NHS Plan and expansion of workforce for the NHS (Department of Health)^23^	The NHS Plan (2000) resulted in the expansion of the workforce for the NHS and included NHS dentistry resulting in a drive to increase the number of dentists working for the NHS.
2001	DofH Code of ethical conduct for international recruitment of health professionals (Department of Health)	To ensure adequate workforce to implement the NHS plan, a key policy decision was to recruit overseas qualified health professionals and this code was adopted by all NHS trusts. OQDs were recruited in hospitals and dental schools in non-career service grades.
2001	BDS degrees from countries with bilateral agreements (Australia, Singapore, Hong Kong, Singapore and South Africa) stopped being recognised by GDC if obtained after 2001 (Sinclair et al.)^4^	Cessation of bilateral agreements with the countries could be a result of policy changes at a political level due to regime change, at institutional level due to the cost effectiveness of GDC to be able to recognise the degrees granted by Universities in these countries and increase in the number of dental schools established due to liberalisation and part privatisation of dental education in these countries.
2001	International Qualifying Examination (IQE) introduced. A 3-part examination for non-EEA IDGs. Exemption for Part 1 to those with FDS RCS (Mathewson and Rudkin)^29^	This created a pathway for dentists qualifying from non-EEA countries to register in the UK, keeping a steady supply of overseas dentists.
2001	Devolution concordat on Health and Social Care, devolving health care to the four nations (Department of Health)^24^	Devolution of health care management to four nations was intended to give more control to local governments to manage the health needs of their population. For dentistry this would lead to variation in delivery of oral health care, difficulty for registrants to relocate internally. Joint planning of dental health workforce was lacking.
2002	Route to reform: A strategy for primary dental care in Wales (Welsh Assembly Government)^26^	Each of the four nations Wales, England, Scotland and Northern Ireland formed different strategies for the delivery of oral health care and its workforce needs.
2004	Dental workforce review (England) Reform with a bite (Department of Health)^25^	Each of the four nations Wales, England, Scotland and Northern Ireland formed different strategies for the delivery of oral health care and its workforce needs.
2005	Action plan for improving oral health and modernising NHS Dental services in Scotland (The Scottish Executive)^27^	Each of the four nations Wales, England, Scotland and Northern Ireland formed different strategies for the delivery of oral health care and its workforce needs.
2005	EU Directive 2005/36/EC – Mutual recognition of EU health professionals’ qualifications (European Union 2005)^34^	This directive facilitated migration of all health professionals across EU and led to a high number of EEA qualified dentists registering to work in the UK.
2006	IQE suspended (Br Dent J 2006)^29^	Large number of non-EEA qualified dentists applied for these registration examinations which proved to be logistical and financial challenge to the GDC. While the route proved that the UK was an attractive destination country, there was limited capacity in the system to manage the flow.
2006	DCP registration opens – GDC (Professions Complementary to Dentistry Regulations order of the Council 2006) (General Dental Council 2006)^40^	The Dental Care Professionals (DCPs) included dental nurses, hygienists, therapists and technicians to enable skill mix in service provision. This could potentially reduce the reliance on overseas qualified dentists.
2006	NHS dental contracts with UDAs introduced in England and Wales (The NHS Regulations)^39^	Changes to the contracts were not popular and are believed to have led to several dentists leaving the NHS for private sector.
2007–2014	IDGs migrated from Bulgaria, Romania, Spain, Portugal, Greece (Sinclair et al.)^4^	The shortage of dentists in the NHS, especially in certain areas of the country was filled with EEA qualified dentists.
2007	IQE replaced with ORE (Br Dent J 2007)^30^	A more streamlined ORE examination replaced the IQE and was conducted by a consortium of dental schools.
2010	WHO Global code of practice on international recruitment of health professionals (WHO)^15^	Adoption of this Code marks a significant achievement for the WHO, more importantly recognising the movement of health professionals, their human rights and creates a global framework for ethical recruitment.
2010	LDS RCS examination changed to ORE format (Royal College of Surgeons England)^31^	The Royal College of Surgeons England created an additional route for overseas dentists to register resulting in more number of non-EEA qualified dentists registering in the UK.
2013	The National Health Service Performer Lists Regulations 2013. (The National Health Service Performers Lists Regulations 2013) England^41^	This was a regulatory framework for managing medical, dental and ophthalmic performers who performed in the primary care services and entrusted the responsibility for managing the performers lists to NHS England as the commissioner of primary care services. The application process to this list varied for graduates of UK, EEA and non-EEA.
2016	English Language requirement by GDC for all applicants to the Register (General Dental Council 2016)^42^	There was always a requirement for all non-EEA registrants to demonstrate their English language competency and exceptions to EEA graduates. This change in the regulations made it possible for GDC to apply it to all registrants.

The timelines of events in dentistry and health care with potential to impact IDG migration.

Meanwhile in Europe, in 2000, the Immigration Order 2000,^32^ allowed short-term migration of EEA citizens and at that point, 20% of EEA registrants in UK had qualified from Germany, Spain, Sweden and Ireland (Table 3). New countries joined the EU in 2004, 2007 and 2013 including Poland, Bulgaria, and Romania and the EU Directive 2005/36/EC, granting mutual recognition of professional qualifications across the EU, was introduced.^33^ In addition, economic recession affected several countries notably Greece, Spain and Germany in 2008, which led to dentists exploring opportunities to practice across Europe.^34,35^ Finally in June 2016, the UK population voted to leave the EU (Brexit) and formally left EU on 31st January 2020^36,37^ (Table 3).

**Table 3:** Timeline of immigration and other events impacting IDGs’ migration and their professional integration.

Year	Events
2000	Immigration Order 2000—Short-term migration of EEA citizens allowed (The Immigration (Designation of Travel Bans) (Amendment) Order 2000)^32^
2002	Highly Skilled Migrant Programme (HSMP) allowed non-EEA IDGs to apply to work in the UK (The Joint Committee on Human Rights)^43^
2004	Expansion of the EU to include Cyprus, Czech Republic, Estonia, Hungary, Latvia, Lithuania, Malta, Poland, Slovakia, and Slovenia (European Union 2011)^33^
2005	Point system for non-EEA workers under ‘Controlling our borders, making migration work for Britain’ (Home Office)^44^
2006	The immigration Asylum and Nationality Act 2006, prevented IDGs on a 4 year ‘Permit free training’ visa from applying for a permanent residency (Home Office 2006)^45^
2007	EU expansion to include Bulgaria and Romania with restrictions on general migration (European Union 2011)^33^
2008	HSMP ends. Tier 1, 2, 5 of points-based system for non-EEA nationals, impacted on IDGs who were in the country as dependants of other HSMP (The Joint Committee on Human Rights)^43^
2008	Eurozone and global financial crisis (BBC News 2019)^35^
2013	EU expansion to include Croatia (European Union 2017)^33^
2016	UK voted to leave the EU—Brexit (Prime Minister’s Office)^36^
2020	UK leaves EU and enters transition period on 31st January 2020 (BBC News)^37^

Table correlating immigration rules and regulations that potentially impacted IDG migration.

Public General Acts form the largest category of legislation, in principle affecting the public general law applying to everyone across the entire United Kingdom (or at least to one or more of its constituent countries of England, Northern Ireland, Scotland, or Wales). https://www.legislation.gov.uk/ukpga.

UK Statutory Instruments (SIs) are a form of legislation which allow the provisions of an Act of Parliament to be subsequently brought into force or altered without Parliament having to pass a new Act. They are also referred to as secondary, delegated or subordinate legislation. (https://www.parliament.uk/documents/commons-information-office/l07.pdf).

## Discussion

Analysis of UK registration trends over the past two decades reveals that the number of dentists has increased, with IDGs from both the EEA and ORE/IQE routes being the primary contributors and suggesting that the UK is an attractive destination for IDGs such that the total number of IDGs has increased from 18% to 28% of the registrant body. Ecological analysis of workforce trends, against key policies, suggested that peaks appear to be associated with key events relating to active international recruitment and border agency policies.

### International recruitment

Modernising NHS Dentistry: Implementing the NHS Plan strategy,^23^ and subsequent reviews into the primary dental care workforce,^25–28^ resulted in active recruitment of IDGs as one of the policy measures to increase dentist supply in order to meet population needs and demands for access to care. Expansion of the EU in 2004, 2007 and 2013 and the EU directive allowing mutual recognition of dental qualifications in the EEA region,^33,35^ together with the IQE/ORE^29,30^ and the recognition of the LDS RCS examination (2010)^31^ facilitated this increase. The countries of primary qualification of the IDGs registered in the UK have also changed significantly, potentially due to the cessation of traditional bilateral agreements (largely based on commonwealth relationships),^4^ active recruitment of IDGs from new EU countries,^33^ greater global mobility of health professionals including IDGs,^38^ changes to the UK immigration, health care delivery and wider political changes in the UK and globally, including in Europe (Tables 1–3).

Overall, our findings suggest that the UK dentistry has become increasingly reliant on an international workforce with a notably higher percentage of IDGs joining the register than UK graduates for a 6-year period (2004–2010). During 2006, several changes were introduced in dentistry which potentially influenced the composition of the registrants. The NHS England UDA-linked contracts may have increased the UK dentists leaving the register.^39^ Registration of dental care professionals (DCPs)^40^ and suspension of IQE^30^ may have led to employers actively recruiting from the EEA. However, the Performer List Regulations (2013),^41^ which created different pathways for UK, EEA and non-EEA dentists to obtain a performer number to work in the NHS in England and the English language assessments introduced in 2016 to protect patients from poor communication,^42^ potentially created additional barriers for IDGs to work in the NHS.

National immigration policies after 2000 facilitated the migration of the EEA health workforce, whilst for the non-EEA workforce the policies were inconsistent.^10,32,43,44^ The highly skilled migrant programme (2002–2008),^43^ together with permit free training visas (until 2006),^44^ enabled non-EEA IDGs to work in secondary care hospital training posts after which the points-based system was introduced (2008),^44^ which has made it more difficult for non-EEA IDGs to work in the UK (Table 3).

### Implications of Brexit and COVID-19

The full impact of Brexit on IDGs migration is yet to be determined and will probably never be fully understood, given the global challenges of the COVID-19 pandemic which is causing major disruption to general health and oral health care systems. Considering that 16% (*n* = 6,761) of the dentists working in the UK in 2019 were from the EEA, the process of how the UK withdraws from the EU will have implications for the supply of dentists in the areas of greatest need nationally.^45^ The potential impact could include a number of factors. First, a reduction in the EEA IDGs who are the main source of the overseas supply of dentists to the UK (Figs. 1 and 2). Second, a reduction in the number of British citizens migrating to the EEA region to obtain a dental qualification, or returning to the UK to practice may further reduce the total numbers.^46^ Third, the absence of bilateral agreements with countries within or outside the EU, when the EU Directives expire, leaves ORE as the only route to registration. ORE is resource intensive, has limited capacity, a high attrition rate and has been suspended indefinitely due to the COVID-19 pandemic.^47^ In this regard Ireland is an exception as it has always had a special agreement under the Common Travel Area since 1921, allowing health workforce movement, irrespective of its relationship to the EEA, a relationship that was reaffirmed in 2019.^48^ Fourth, dentists who are successful in passing ORE are struggling to find jobs in the NHS due to the lack of practices willing to support them through the ‘performers list validation by equivalence’ process to obtain a performer number.^49^ Therefore registrant numbers are not translated to actual numbers of dentists working in the NHS. Fifth, the message that immigrants are ‘not welcome’ may potentially reduce the migration of both EEA and non-EEA IDGs^49,50^ to the UK resulting in difficulties recruiting dentists especially in the areas where UK graduates are reluctant to work.

Post Brexit, the Government has advised all EU NHS staff to apply through the UK settlement status scheme and advised employers not to alter their contracts. Professional regulators including the GDC have been asked to provide reassurances to EEA IDGs that their qualifications will continue to be recognised,^51^ however, application and interpretations of the above schemes may limit the employment of EU dentists.^52^ During the transition period the legislation requires that the EEA route to registration is kept open and that EU qualifications continue to be recognised, although there are suggestions that Brexit has made the EEA dentists feel unwelcome in the UK.^50^ Registrant numbers (Fig. 2) clearly show a downward trend from 2015 onwards, with more IDGs leaving than joining until 2017, although numbers have plateaued thereafter.

### Strengths and limitations of the data presented

The GDC registrant data, maintained since 1956,^53^ does not represent the true workforce capacity as the total number of registrants does not equate to the full-time equivalent of dentists involved in providing clinical service. Nor is it a record of all the IDGs in the UK or of the non-registered IDGs contributing to the academia and the NHS. There may be a number of IDGs working in the dental profession in roles that do not require GDC registration or are registered as DCPs. The GDC summary data are a snapshot of the number of registrants at a given point in time. The GDC Annual Reports lack the detailed breakdown of the data needed for research into migration of IDGs and information had to be obtained through several FOI requests. Recent changes to the GDC’s data management systems meant that some source country data (2015–2018) were not retrievable through an FOI. An online platform similar to the GMC Data Explorer, an interactive tool that provides the most recent live data on age, gender, country of qualification and area of distribution, based on speciality may provide an open, transparent and accessible platform to obtain data to facilitate research.^54^

### Implications and future research

Recent reports of difficulties in recruiting dentists to work for the NHS in certain areas,^55^ may be partly due to UK graduates opting to work less hours and more flexibly, an increase in female dentists with parental leave requirements, increased time spent on paperwork, portfolio careers, choosing the private sector due to the decrease in NHS remuneration^56^ and burnout.^57^ The number of dental practices in England reporting difficulty in recruiting dentists had increased from 49% in 2016 to 66% in 2017.^58^ In the UK there is little information on the movement of dentists across the four nations and the bodies involved in dental workforce planning have highlighted the need for monitoring migration of dentists to each country. However, workforce considerations largely focus on state provided dental care and there is limited data regarding dentists working in the private sector to enable planning for the whole workforce. The reasons for an increase in female migration and their migration motivations is currently unknown. The most likely explanations are; first, that the majority of graduates in many European countries, particularly eastern Europe are female.^46,59^ Second, they are migrating as dependants of other highly skilled migrants as a result of the wider dependency of the UK labour market in other sectors.^60^ Third, the UK may be considered a safer country for females to live and supportive of female careers. However, evidence suggests that gender may play a significant role in IDGs’ career trajectories as females could face greater challenges than their male counterparts, as reported by female nurses and doctors who migrated to the UK.^60,61^

Research into the retention of dentists in the workforce is required as reduction of income, increased burden of litigation, and a deterioration in working conditions are known to push health professionals including immigrants to emigrate further to countries, such as Australia, New Zealand, the USA and Canada and away from the NHS.^13,60^ Migration of dental graduates is a global concern as it leads to unequal distribution of the dental workforce, at a time when globally diseases including dental caries, periodontal disease and oral cancer are increasing.^14,62^ There is evidence that there are deficiencies in the number of dentists trained in some regions of the world, while in others there is an urban surplus leading to unemployment, unwillingness to work in the state commissioned dental services and a mismatch of dental education to the disease profiles in the countries.^12,13,60^ Monitoring the internal and external migration of dentists requires collection of data on inflows and outflows of registrants in the UK including information on the country of primary qualification, country of origin, age, gender, ethnicity, postcode of work location and sector worked to understand the patterns of internal and external migration of dentists.^60^ COVID-19 pandemic has severely restricted national and international travel and created intense pressure on health care systems across the world. Oral health care provision has been minimal to non-existent in several parts of UK since middle of March 2020. IDGs working in UK may have decided to return home before travel restrictions applied or post COVID-19, there may be influx of IDGs into UK looking for opportunities. Although the route to entry for EEA dentists exists, the route for non-EEA dentists is suspended for foreseeable future.^47^ Hundreds of non-EEA dentists who have passed Part 1 of the ORE, waiting to take Part 2 may be facing stagnation of their career plans. While the future of dental workforce supply is uncertain, there are uncertainties of the viability of dental practices too. There is a need for more research into dental workforce migration and the impact of international recruitment, particularly during and post pandemic, in support of delivering access to dental care.

## Conclusion

Health and workforce legislation and policy over the past two decades appear to have facilitated the migration of IDGs to the UK. UK dentistry has become increasingly dependent on IDGs, particularly dentists qualifying from the EEA who are more likely to be impacted by the United Kingdom’s Brexit plans. More research into global, national and international migration of dentists, their migration motivations and professional integration experiences are needed to plan recruitment and retention of dentists and improve access to dental care in the UK.
